# Development of a HPLC-MS/MS Method to Assess the Pharmacokinetics and Tumour Distribution of the Dimethylarginine Dimethylaminohydrolase 1 Inhibitors ZST316 and L-257 in a Xenograft Model of Triple-Negative Breast Cancer in Mice

**DOI:** 10.3390/molecules28248056

**Published:** 2023-12-13

**Authors:** Tommaso Ceruti, Roberta Frapolli, Carmen Ghilardi, Alessandra Decio, Giulia Dellavedova, Sara Tommasi, Massimo Zucchetti, Arduino A. Mangoni

**Affiliations:** 1 Laboratory of Cancer Pharmacology, Department of Oncology, Instituto di Ricerche Farmacologiche Mario Negri IRCCS, 20156 Milan, Italy; tommaso.ceruti@marionegri.it (T.C.); roberta.frapolli@marionegri.it (R.F.); massimo.zucchetti@marionegri.it (M.Z.); 2 Laboratory of Cancer Metastasis Therapeutics, Department of Oncology, Instituto di Ricerche Farmacologiche Mario Negri IRCCS, 20156 Milan, Italy; carmen.ghilardi@marionegri.it (C.G.); alessandra.decio@tiscali.it (A.D.); giulia.dellavedova@guest.marionegri.it (G.D.); 3 Discipline of Clinical Pharmacology, College of Medicine and Public Health, Flinders University, Bedford Park, SA 5042, Australia; sara.tommasi@sa.gov.au; 4 Department of Clinical Pharmacology, Flinders Medical Centre, Southern Adelaide Local Health Network, Bedford Park, SA 5042, Australia

**Keywords:** ZST316, L-257, dimethylarginine dimethylaminohydrolase 1, DDAH1 inhibitors, triple-negative breast cancer, pharmacokinetics, xenograft models, HPLC-MS/MS

## Abstract

We describe the development and validation of an HPLC-MS/MS method to assess the pharmacokinetics and tumour distribution of ZST316, an arginine analogue with inhibitory activity towards dimethylarginine dimethylaminohydrolase 1 (DDAH1) and vasculogenic mimicry, and its active metabolite L-257 in a xenograft model of triple-negative breast cancer (TNBC). The method proved to be reproducible, precise, and highly accurate for the measurement of both compounds in plasma and tumour tissue following acute and chronic (five days) intraperitoneal administration of ZST316 (30 mg/Kg daily) in six-week-old severe combined immunodeficiency disease (SCID) mice inoculated with MDA-MB-231 TNBC cells. ZST316 was detected in tumour tissue and plasma after 1 h (6.47 and 9.01 μM, respectively) and 24 h (0.13 and 0.16 μM, respectively) following acute administration, without accumulation during chronic treatment. Similarly, the metabolite L-257 was found in tumour tissue and plasma after 1 h (15.06 and 8.72 μM, respectively) and 24 h (0.17 and 0.17 μM, respectively) following acute administration of ZST316, without accumulation during chronic treatment. The half-life after acute and chronic treatment ranged between 4.4–7.1 h (plasma) and 4.5–5.0 h (tumour) for ZST316, and 4.2–5.3 h (plasma) and 3.6–4.9 h (tumour) for L-257. The results of our study demonstrate the (a) capacity to accurately measure ZST316 and L-257 concentrations in plasma and tumour tissue in mice using the newly developed HPLC-MS/MS method, (b) rapid conversion of ZST316 into L-257, (c) good intra-tumour penetration of both compounds, and (d) lack of accumulation of both ZST316 and L-257 in plasma and tumour tissue during chronic administration. Compared to a previous method developed by our group to investigate ZST316 in plasma, the main advantages of the new method include a wider range of linearity which reduces the need for dilutions and the combined assessment of ZST316 and L-257 in plasma and tumour tissue which limits the required amount of matrix. The new HPLC-MS/MS method is useful to investigate the in vivo effects of ZST316 and L-257 on vasculogenic mimicry, tumour mass, and metastatic burden in xenograft models of TNBC.

## 1. Introduction

Isoform 1 of the enzyme dimethylarginine dimethylaminohydrolase (DDAH1) is primarily responsible, unlike isoform 2 (DDAH2), for the metabolism of the endogenous inhibitors of nitric oxide (NO) synthesis, asymmetric N^G^,N^G^-dimethyl-L-arginine (ADMA) and NG-monomethyl-L-arginine (NMMA), into L-citrulline and dimethylamine [1,2,3,4,5,6,7,8]. Whilst upregulation of DDAH1 has been originally investigated as a therapeutic strategy to reduce ADMA and NMMA concentrations in conditions characterized by impaired NO synthesis, e.g., atherosclerosis and cardiovascular disease [4,9,10,11], more recently studies have also focused on DDAH1 inhibition to counteract the negative effect of excessive local and/or systemic NO concentrations in other disease states, e.g., cancer and sepsis [12,13,14,15,16,17,18,19,20,21]. Specifically, arginine analogues with DDAH1 inhibitory effects synthesized by our group have been shown to suppress the capacity of triple-negative breast cancer (TNBC) cell lines to undergo vasculogenic mimicry, a critical driver of cancer cell dissemination, metastasis, and adverse outcomes in patients with TBNC and other types of cancer [22,23,24,25,26,27,28,29].

The pharmacokinetics and safety profile of the most promising DDAH1 inhibitors in our series, compounds ZST316 and ZST152, have been recently investigated in four-week-old Friend leukemia virus B (FVB) mice after oral, intravenous, and intraperitoneal administration. In this study, the most potent DDAH1 inhibitor, ZST316 (Ki: 1 μM [22], subsequently revised to 261 nM using a more performant assay [30], personal data) exhibited a favourable pharmacokinetic profile and excellent tolerability [31]. Notably, further analyses also led to the identification of several urinary metabolites of ZST316, including the known DDAH1 inhibitor, compound L-257 [16,18,32]. This observation suggests that the conversion of ZST316 into L-257 could mediate, at least in part, the effects of ZST316 on DDAH1 activity and vasculogenic mimicry in vivo.

In order to further investigate this issue, we report the development and validation of a new HPLC-MS/MS method for the combined measurement of ZST316 and L-257 in plasma and tumour tissue, assessing the pharmacokinetics of both molecules following acute and chronic treatment with ZST316 in a xenograft model of TNBC.

## 2. Results

### 2.1. HPLC-MS/MS

**Figure 1:**
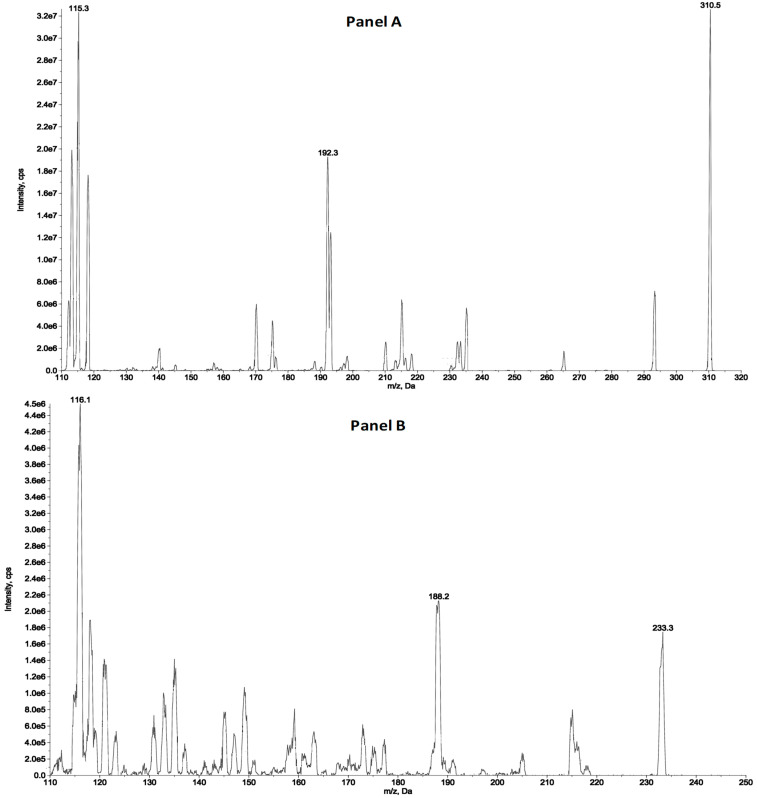
SRM fragmentation pattern of ZST316 (**A**) and L-257 (**B**) obtained by tandem mass spectrometry.

**Figure 2:**
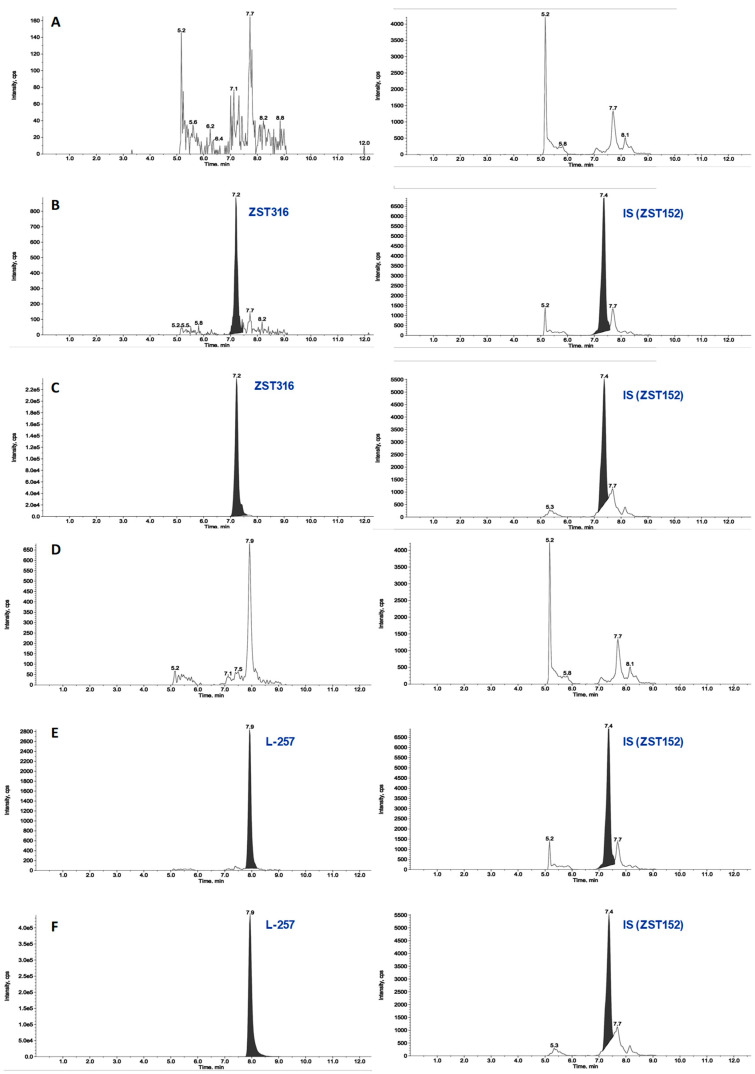
Representative chromatograms of a blank plasma sample (panel (**A**) for ZST316 and (**D**) for L-257), LOQ of ZST316 with IS (panel (**B**)), an unknown study plasma sample analysed for ZST316 (panel (**C**)), LOQ of L-257 with IS (panel (**E**)), and an unknown study plasma sample analysed for L-257 (panel (**F**)). The measured concentration corresponded to 2200 ng/mL and 1710 ng/mL for ZST316 and L-257, respectively.

**Figure 3:**
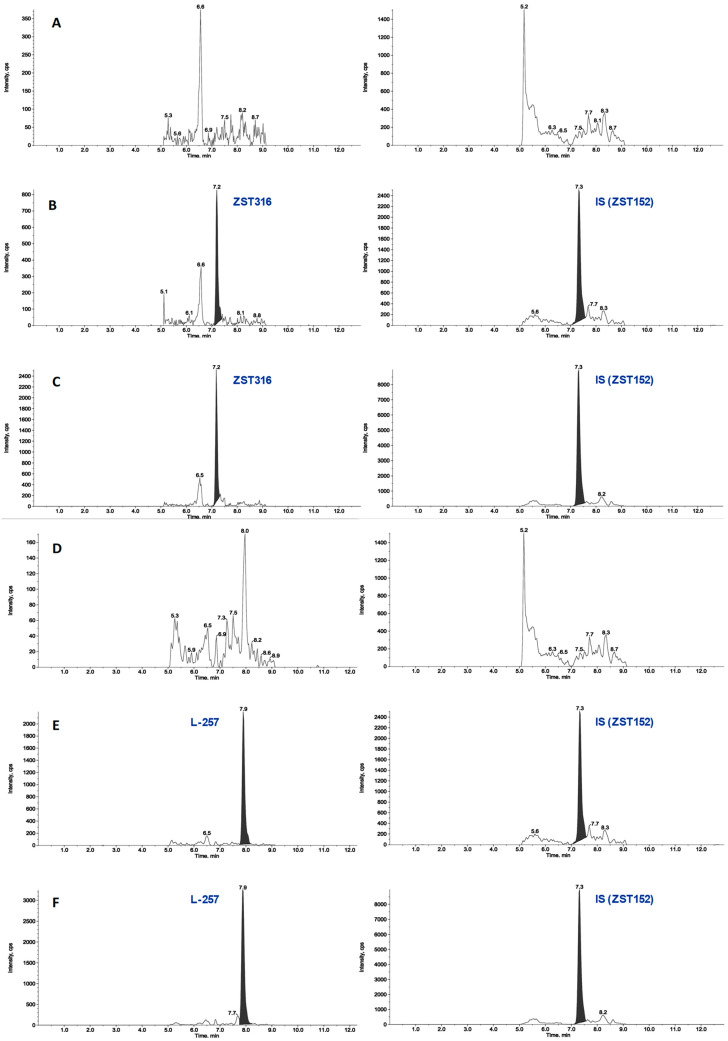
Representative chromatograms of a blank tumour sample (panel (**A**) for ZST316 and (**D**) for L-257), LOQ of ZST316 with IS (ZST152, panel (**B**)), unknown study tumour sample analysed for ZST316 with IS (ZST152, panel (**C**)), LOQ of L-257 with IS (panel (**E**) and unknown study tumour sample analysed for L-257 with IS (panel (**F**)). The measured concentration corresponded to 37.3 ng/g and 30.7 ng/g for ZST316 and L-257, respectively.

The detection and quantification of the analytes were carried out by tandem mass spectrometry (MS/MS) following the following transitions: 310.5 > 115.3 and 310.4 > 192.3 *m*/*z* as quantifier and qualifiers ions for ZST316, respectively and 233.3 > 116.1 and 233.3 > 188.2 as quantifier and qualifiers ions for L-257, respectively. The detailed selected reaction monitoring (SRM) reporting the full fragmentation pattern of ZST316 and L-257 obtained by MS/MS is shown in Figure 1. Figure 2 and Figure 3 reports chromatograms including blank plasma and blank tumour samples, the lower limit of quantitation (i.e., 5 ng/mL and 25 ng/g), and unknown study samples taken at 1 and 24 h after acute treatment for plasma and tumour, respectively.

#### 2.1.1. Plasma Sample Analysis

A quantitation method for ZST316 was previously established [31]. Here, we set up, validated, and applied the method for the combined measurement of ZST316 and L-257. The limit of detection (LOD) of the method was 2 ng/mL for both ZST316 and L-257 (Supplementary Table S1). Lower concentrations (i.e., 1.5 ng/mL) were indistinguishable from the background noise (S/N < 3). We established a LOQ of 5 ng/mL for L-257, in line with that of ZST316 [31], with precision of 5.2% and accuracy of 107.8% (*n* = 5). The linearity of the method for L-257 was acceptable over the concentration range 5.0–1000.0 ng/mL, with a coefficient of determination (R^2^) of 0.996.

The combined method proved to be precise and accurate as shown by the analysis of three different concentrations of QC plasma samples of ZST316 and L-257 (Table 1). Both ZST316 and L-257 were stable in frozen plasma for at least one month at −20 °C (Table 2). There was no chromatographic carryover effect observed injecting a blank sample after the highest standard calibration point. 

Considering the precision (range 5.2–9.9%) and accuracy (range 95.5–107.8%) values found for QCs and LOQ, all falling within the parameters defined by the main international guidelines [33,34], the method developed for the combined measurement of ZST316 and L-257 in plasma is considered suitable for preclinical pharmacokinetic investigations. 

#### 2.1.2. Tumour Tissue

The method was linear in the tested standards among the concentration range of 25.0–5000.0 ng/mL for both analytes, as demonstrated by the mean determination coefficient (R^2^) of 0.997 and 0.994 for ZST316 and L-257, respectively. 

The recovery was high and consistent at all the QC concentrations of ZST316 and L-257 (Table 3 and Table 4). We established a LOQ of 25 ng/g for both the analytes in tumour, with precision of 3.3 and 4.6% and an accuracy of 102.7 and 110.5, for ZST316 and L-257 (*n* = 5). The LOD of the method in tumour tissue was estimated to be 1/3 of the LOQ (8.3 ng/g) as direct determination was prevented by insufficient control matrix.

The method was reproducible, precise, and accurate as shown by the analysis of QC homogenate tumour samples containing both ZST316 and L-257 (Table 5 and Table 6). No chromatographic carryover effect was observed injecting a blank sample after the highest standard calibration point.

Considering the high and consistent recovery, the precision (range 3.3–8.5%) and accuracy (range 101.8–110.5%) values found for QCs and LOQ, all falling within the parameters defined by the main international guidelines, we believe that the method developed for the combined measurement of ZST316 and L-257 in tumour is suitable for preclinical pharmacokinetic investigations. 

### 2.2. Pharmacokinetics and Tumour Distribution

#### 2.2.1. Acute Treatment with ZST316

**Figure 4:**
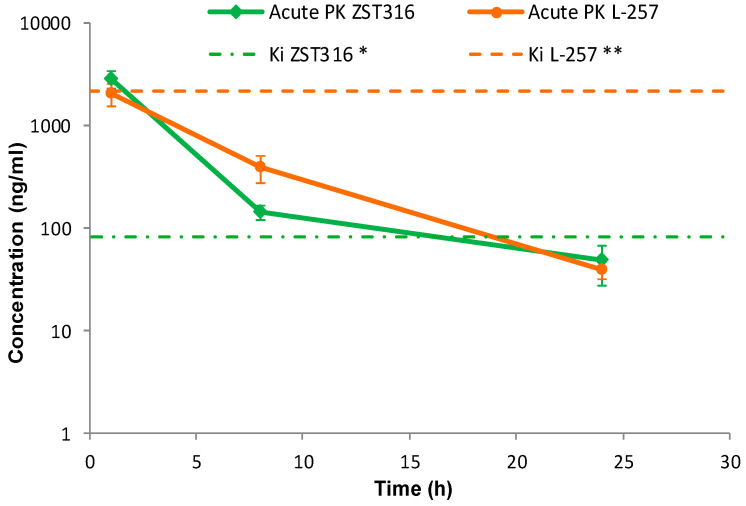
Plasma pharmacokinetics of ZST316 and L-257 after acute administration of ZST316. Dotted lines represent the Ki values (* 261 nM = 80.3 ng/mL; ** 7 μM = 1625 ng/mL; raw data are presented in Supplementary Tables S2 and S3).

ZST316 was detected in plasma at a mean concentration of 2.79 μg/mL (9.01 μM) one hour after acute intraperitoneal administration. The drug was rapidly cleared with an estimated elimination half-life of 4.4 h and was still detectable after 24 h at a mean concentration of 0.05 μg/mL (0.16 μM), as shown in Figure 4 and Table 7. The active metabolite L-257 was measurable in plasma at a concentration of 2.02 μg/mL (8.72 μM) one hour after acute intraperitoneal administration of ZST316. The estimated half-life of the drug was 4.2 h and ZST316 was detectable up to 24 h at a mean concentration of 0.04 μg/mL (0.17 μM; Figure 4 and Table 7).

**Figure 5:**
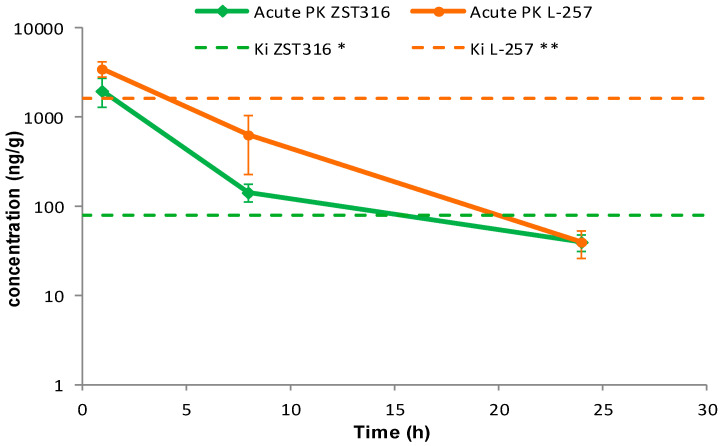
Tumour distribution of ZST316 and L-257 after acute administration of ZST316. Dotted lines represent the Ki values (* 261 nM = 80.3 ng/mL; ** 7 μM = 1625 ng/mL; raw data are presented in Supplementary Tables S4 and S5).

Intra-tumour ZST316 was detected at a concentration of 2.0 μg/g (6.47 μM) one hour after acute intraperitoneal administration. The drug was rapidly cleared with an estimated elimination half-life of 4.5 h. The drug was still detectable after 24 h at a mean tumour concentration of 0.04 μg/g (0.13 μM), as reported in Figure 5 and Table 7. Intra-tumour L-257 was detected at a concentration of 3.5 μg/g (15.06 μM) one hour after acute intraperitoneal administration of ZST316. L-257 was rapidly cleared with an estimated elimination half-life of 3.6 h. The metabolite was detectable after 24 h at a mean concentration of 0.04 μg/g (0.17 μM; Figure 5 and Table 7).

#### 2.2.2. Chronic Treatment with ZST316

**Figure 6:**
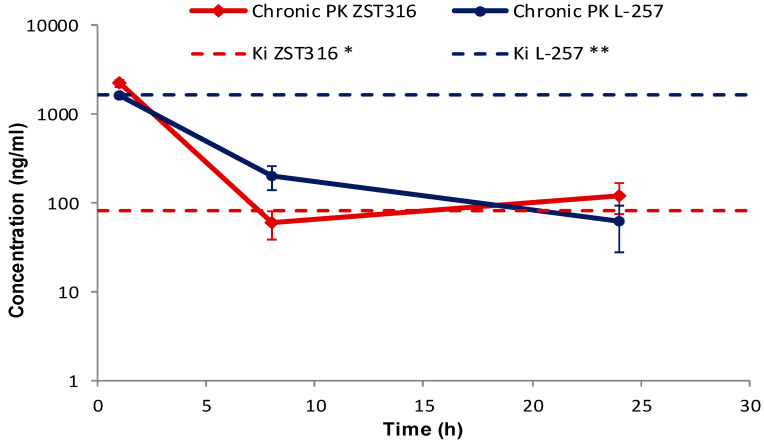
Plasma pharmacokinetics of ZST316 and L-257 after chronic administration of ZST316. Dotted lines represent the Ki values (* 261 nM = 80.3 ng/mL; ** 7 μM = 1625 ng/mL; raw data presented in Supplementary Tables S6 and S7).

ZST316 was detected in plasma at a mean concentration of 2.22 μg/mL (7.19 μM) one hour after chronic intraperitoneal administration, then cleared from plasma with a half-life of 7.1 h. The drug was still detectable after 24 h at a mean plasma concentration of 0.12 μg/mL (0.39 μM; Figure 6 and Table 7). The metabolite L-257 was measurable in plasma at a concentration of 1.60 μg/mL (6.91 μM) one hour after chronic administration of ZST316. Estimated half-life was 5.3 h. L-257 was still detectable in plasma up to 24 h at a mean concentration of 0.06 μg/mL (0.26 μM; Figure 6 and Table 7).

**Figure 7:**
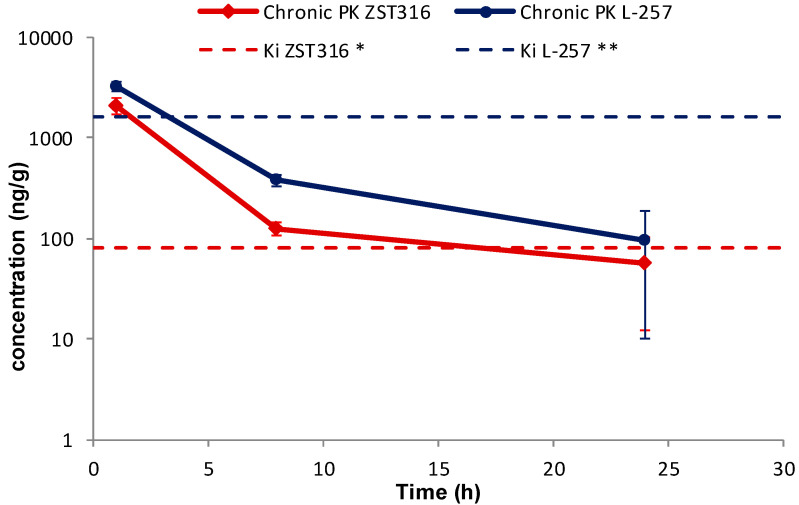
Tumour distribution of ZST316 and L-257 after chronic administration of ZST316. Dotted lines represent the Ki values (* 261 nM = 80.3 ng/mL; ** 7 μM = 1625 ng/mL; raw data presented in Supplementary Tables S8 and S9).

Intra-tumour ZST316 was detected at a concentration of 2.09 μg/g (6.75 μM) one hour after chronic intraperitoneal administration. The drug was rapidly cleared with an estimated elimination half-life of 5.0 h. The drug was still detectable after 24 h at a mean tumour tissue concentration of 0.06 μg/g (0.18 μM; Figure 7 and Table 7). Intra-tumour L-257 was detected at a concentration of 3.24 μg/g (13.94 μM) 1 h after chronic intraperitoneal administration of ZST316. L-257 was rapidly cleared with an elimination half-life of 4.9 h. The metabolite was still detectable after 24 h at a mean concentration of 0.10 μg/g (0.30 μM; Figure 7 and Table 7).

No accumulation of ZST316 was observed after chronic treatment, with similar plasma areas under the curve (AUC) observed at day 1 (15.3 μg/mL·h) and day 5 (12.4 μg/mL·h; Table 7). The acute and chronic tumour AUC values were also similar (11.4 and 11.8 μg/g·h; Table 7). There was also no accumulation of the metabolite L-257 after chronic treatment, with similar plasma areas under the curve (AUC) observed at day 1 (14.1 μg/mL·h) and day 5 (10.3 μg/mL·h; Table 7). The acute and chronic tumour AUC values were also similar (23.9 and 20.3 μg/g·h; Table 7).

## 3. Materials and Methods

### 3.1. Compounds

ZST316, L-257, and ZST152 (Internal Standard, IS) were provided by Flinders University (Adelaide, Australia). The compounds were dissolved in bi-distilled water, according to the instructions from the provider, for method development and pharmacokinetic analysis. ZST316 was dissolved in sterile water prior to acute and chronic intraperitoneal administration in mice (S.A.L.F. S.p.A., Laboratorio Farmacologico, Bergamo, Italy). 

### 3.2. Pharmacokinetic Study

Six-week-old female severe combined immunodeficiency disease (SCID) mice (*n* = 24; Charles River Laboratories Italia S.r.L., Calco, Lecco, Italy), were housed under rigorous standards and handled under pathogen-free conditions in the Animal Care Facilities of the Mario Negri Institute for Pharmacological Research (Milan, Italy). 

Two ×10^6^ (in 10 μL) MDA-MB-231 TNBC cells were transplanted into the mammary fat pad. Tumour growth was regularly monitored with a calliper and treatment with ZST316 was given when the tumour volume reached 400–500 mm^3^. Mice received either a single dose (acute treatment) or a daily dose of ZST316 30 mg/Kg intraperitoneally for five days (chronic treatment). Chronic treatment was well tolerated, and no signs of toxicity were recorded.

Plasma and tumour tissue (*n* = 3 mice/each time point) were sampled at 1, 8 and 24 h after treatment (on day 5 for the chronic treatment). To collect plasma, mice were anesthetized by isoflurane, the blood was drained from the retro-orbital plexus into heparinised tubes and centrifuged for 10 min at 4000× *g* at 4 °C. Tumours were collected and immediately frozen. Plasma and tumour samples were stored at −20 °C until analysis.

### 3.3. Analytical Method Development

#### 3.3.1. Preparation of Standard and Quality Control Plasma Samples

Plasma from control mice (90 μL) was spiked with 5 μL of ZST316 and L-257 working solutions prepared in the range 100–20,000 ng/mL, generating seven plasma calibration standards in the range 5.0–1000 ng/mL. The calibration curve included a blank and a zero blank standard plasma sample (processed with IS). To prepare quality control (QC) samples, three fractions of plasma were mixed with an appropriate amount of QC working solutions to obtain QC plasma samples at the final concentration of 15, 75, and 250 ng/mL.

#### 3.3.2. Extraction Procedure for ZST316 and L-257 in Plasma

To assay ZST316 and L-257, 10 μL of IS (ZST152 WS: 500 ng/mL) were added to 100 μL of study plasma samples, standards, and QCs in a polypropylene tube. The samples were then added with 10 μL of NH_4_COOH 50 mM, mixed and added with 400 μL CH_3_OH:0.1% HCOOH, vortex mixed again and centrifuged at 13,200 rpm for 10 min at 4 °C. The supernatant was transferred to a 1.5 mL Eppendorf tube, dried under nitrogen flow and reconstituted with 100 μL of mobile phase (MP) A:B (1:1, *v*/*v*). Finally, the samples were centrifuged at 13,200 rpm for 10 min at 4 °C and the supernatant transferred into vials for HPLC-MS/MS analysis.

#### 3.3.3. Stability of ZST316 and L-257 in Frozen Plasma

The stability of ZST316 and L-257 in plasma was assessed by analysing QC samples containing both compounds after prolonged storage in the same conditions of the study samples. 

Stability was determined by analysing 3 QCs per concentration level immediately after the preparation, and 3 QCs per concentration level after one month of storage at −20 °C. The analytes were considered stable at each QC concentration when the freshly prepared samples and the stability test samples differed by no more than 15% from the nominal concentrations.

#### 3.3.4. Preparation of Standard and Quality Control Tumour Samples

Control MDA-MB-231 TNBC cell tumours were homogenized with NH_4_COOH 50 mM (1:4, *w*:*v*). The homogenate (90 μL) was spiked with 5 μL of ZST316 and L-257 working solutions prepared in the range of 100–10,000 ng/mL generating seven tumour calibration standards in the range 25–5000 ng/g. The calibration curve included also a blank and a zero blank standard plasma sample (processed with IS). To prepare QC samples, 90 μL of the homogenate was mixed with an appropriate amount of QC working solutions to obtain QC plasma samples at the nominal concentration of 75, 375, and 1250 ng/g.

#### 3.3.5. Extraction Procedure for ZST316 and L-257 in Homogenate Tumour Study Samples, Standards, and Quality Controls

The procedure was similar to that described for plasma in Section 3.3.2.

#### 3.3.6. Recovery of ZST316 and L-257 in Tumour Tissue

ZST316 extraction efficiency (recovery) was determined by comparing the peak area of the analyte extracted from tumour homogenate in QC samples prepared in quintuplicate at low, medium, and high ZST316 concentrations (75, 375 and 1250 ng/g) with the mean peak area of the analyte measured in the extracted matrix added with the same amount of ZST316 (five replicates for each QC level). 

L-257 recovery was determined by comparing the peak area of the analyte extracted from tumour homogenate in QC samples prepared in quintuplicate at low, medium, and high L-257 concentrations (75, 375 and 1250 ng/g) with the mean peak area of the analyte measured in the extracted matrix added with the same amount of L-257 (five replicates for each QC level).

#### 3.3.7. HPLC-MS/MS Conditions

Reversed-phase chromatography was performed under gradient conditions with separation on a HILIC column, C18, 3 μm, 100 A, 2.1 × 150 mm, Waters (Milford, MA, USA), coupled with a 3 μm, 3.9 × 5.0 mm guard column of the same material at the flow rate of 0.2 mL/min. Gradient features were: 10% mobile phase (MP) A (NH_4_HCO_2_ 10 mM, 10% CH_3_CN, 0.1% HCOOH) and 90% MP B (CH_3_CN, 0.1% HCOOH) as initial constant condition; then to 90% MP A in 6 min and constant condition for 1.5 min. Return to start condition in 0.5 min and conditioning for 5.5 min. Total run time: 13.5 min. Analyte detection and quantification were performed using tandem mass spectrometry (MS/MS) operating in positive ionization mode as previously reported for ZST316 and ZST152 [31]. 

## 4. Discussion

Using a previously developed method, we reported the pharmacokinetic profile of the arginine analogue DDAH1 inhibitors ZST316 and ZST152 in mice, and the observation that urinary metabolites of ZST316 include another known DDAH1 inhibitor, compound L-257 [31]. In this study, we describe the development of an accurate and reproducible bioanalytical method for the combined assessment of ZST316 and L-257 in plasma and tumour tissue. A description of the similarities and differences between the old and the new method is provided in Supplementary Table S10. The main advantages of the new method are that a wider range of linearity reduces the need for dilutions and that the combined assessment of ZST316 and L-257 allows a more comprehensive pharmacokinetic analysis and limits the required amount of matrix (Supplementary Table S10). 

The new method was successfully applied to assess the pharmacokinetics and for the first time the tumour distribution of both compounds following acute and chronic intraperitoneal treatment with ZST316 in a xenograft model of TNBC using the established cell line MDA-MB-231 [35,36,37]. The results of these assessments indicate that ZST316 is rapidly converted into L-257 in vivo, possibly by amidases, and that both compounds share a similar pharmacokinetic profile (Supplementary Figure S1) [18,38,39,40]. Notably, both compounds rapidly penetrate the tumour tissue and are still detectable 24 h after administration of ZST316. Furthermore, there was no evidence of plasma or tumour accumulation for both ZST316 and L-257 during chronic treatment with ZST316.

The preferential tumour vs. plasma localization observed for the ZST316 metabolite and DDAH1 inhibitor L-257 (as indicated by the high AUC_tum_/AUC_pl_ ratio; Table 7) raises the possibility that the reported in vitro effects of compound ZST316 on vasculogenic mimicry might also depend on its conversion to L-257 in vivo [20,23]. However, it is also important to emphasise that the observed intra-tumour concentrations of L-257 remained above the IC_50_ [32] and the Ki values for DDAH1 inhibition [18,22] for a relatively short period of time, an estimated 4 and 3.5 h after acute and chronic ZST316 treatment, respectively, when compared to the time above the IC_50_ and Ki values for ZST316, 15.5 and 16.5 h after acute and chronic ZST316 treatment (Figure 5 and Figure 7) [22,30]. 

The pharmacokinetic and tumour distribution data reported in this study allow the optimal planning of activity studies in xenograft models of TNBC. Specifically, the plasma and tumour exposure which we determined to be 16–20 h at concentrations ≥ ki allows us to foresee daily treatments with ZST316 for relatively long periods of time, also given the good tolerability observed in this and in a previous study [31]. 

Considering the positive data originated by the combined exposure of the two agents, the newly developed HPLC-MS/MS method appears particularly useful for investigating the effects of ZST316 treatment on vasculogenic mimicry, tumour growth, and metastatic burden in xenograft models of TNBC and whether such effects are at least partially mediated by the active metabolite L-257. 

## 5. Conclusions

The development and validation of a new HPLC-MS/MS method for the combined determination of the DDAH1 inhibitor ZST316 and its active metabolite L-257 allowed a comprehensive characterization of the pharmacokinetics and tumour distribution of both compounds after acute and chronic treatment with ZST316. This method appears to be particularly useful for investigating the effects of pharmacological inhibition of DDAH1 on vasculogenic mimicry, tumour growth, and metastatic burden in xenograft models of cancer, specifically TNBC. 

## Figures and Tables

**Table 1:** Precision and accuracy results for the assay of ZST316 and L-257 in plasma.

	ZST316			L-257		
Actual concentration (ng/mL)	15.0	75.0	250.0	15.0	75.0	250.0
Mean concentration (ng/mL)	15.1	71.6	257.7	14.7	75.2	259.3
Accuracy (%)	100.7	95.5	103.1	98.0	100.3	103.7
Precision (%)	7.6	9.9	7.8	2.4	2.1	5.3
# N	3	3	3	3	3	3

**Table 2:** Stability assay of ZST316 and L-257 in plasma samples.

	ZST316						L-257					
	Fresh (T0)			Stability −20 °C			Fresh (T0)			Stability −20 °C		
Actual value (ng/mL)	15.00	75.00	250.00	15.00	75.00	250.00	15.00	75.00	250.00	15.00	75.00	250.00
Found value (ng/mL)	13	68.3	244	14.2	70.7	234.5	16	74.9	265	15.5	77.1	263.5
	12.8	69.2	258	14.6	75.0	234.1	14.9	83	262	15.7	75.5	242.0
	13.6	73	245	14.1	71.5	251.3	16.4	81.8	256	16.3	74.6	273.1
N	3	3	3	3	3	3	3	3	3	3	3	3
Mean	13.1	70.2	249.0	14.3	72.4	240.0	15.8	79.9	261.0	15.8	75.7	259.5
SD	0.42	2.49	7.81	0.24	2.25	9.81	0.78	4.37	4.58	0.37	1.25	15.92
Precision (%)	3.17	3.56	3.14	1.68	3.11	4.09	4.93	5.47	1.76	2.34	1.65	6.14
Accuracy (%)	87.6	93.6	99.6	95.4	96.5	96.0	105.1	106.5	104.4	105.6	101.0	103.8

**Table 3:** Extraction recovery in homogenate of tumour tissue for ZST316.

	QC 75 ng/g	QC 375 ng/g	QC 1250 ng/g
Sample No.	Recovery %		
1	86.5	112.6	95.6
2	81.8	98.0	86.9
3	86.5	98.6	84.9
4	89.5	98.7	82.4
5	77.6	81.7	85.7
N	5	5	5
Mean	84.4	97.9	87.1
SD	4.7	10.9	5.0
CV%	5.5	11.2	5.8

Legend: QC, quality control; SD, standard deviation; CV, coefficient of variation.

**Table 4:** Extraction recovery in homogenate of tumour tissue for L-257.

	QC 75 ng/g	QC 375 ng/g	QC 1250 ng/g
Sample No.	Recovery%		
1	98.4	102.5	94.4
2	104.4	100.1	90.0
3	99.7	91.5	90.8
4	91.0	92.4	90.0
5	76.1	90.0	89.9
N	5	5	5
Mean	93.9	95.3	91.0
SD	11.1	5.6	1.9
CV%	11.8	5.9	2.1

Legend: QC, quality control; SD, standard deviation; CV, coefficient of variation.

**Table 5:** Precision and accuracy results for ZST316 in tumour tissue.

Actual concentration (ng/g)	25.0 (LOQ)	75.0	375.0	1250.0
Mean concentration found (ng/g)	25.7	81.5	382	1281
Accuracy (%)	102.7	108.7	101.8	102.5
Precision (%)	3.3	5.7	8.5	4.1
Number of samples	5	5	5	5

Legend: LOQ, limit of quantification.

**Table 6:** Precision and accuracy results for L-257 in tumour tissue.

Actual concentration (ng/g)	25.0 (LOQ)	75.0	375.0	1250.0
Mean concentration found (ng/g)	27.6	80.2	397	1356
Accuracy (%)	110.5	106.9	105.7	108.5
Precision (%)	4.6	6.8	4.4	3.9
Number of samples	5	5	5	5

Legend: LOQ, limit of quantification.

**Table 7:** Pharmacokinetic parameters of ZST316 and L-257 after acute and chronic intraperitoneal administration of ZST316.

	ZST316 Acute	ZST316 Chronic	L-257 Acute	L-257 Chronic
Plasma AUC_1–24 h_ (μg/mL·h)	15.3 (0.050 μM/mL·h)	12.4 (0.040 μM/mL·h)	14.1 (0.061 μM/mL·h)	10.3 (0.044 μM/mL·h)
Tumour AUC_1–24 h_ (μg/mL·h)	11.4 (0.037 μM/mL·h)	11.8 (0.038 μM/mL·h)	23.9 (0.103 μM/mL·h)	20.3 (0.087 μM/mL·h)
AUC_tum_/AUC_pl_	0.75	0.95	1.7	2.0
C_max_ plasma (μg/mL)	2.79 (9.03 μM)	2.22 (7.18 μM)	2.02 (8.70 μM)	1.60 (6.89 μM)
C_max_ tumour (μg/g)	2.00 (6.47 μM)	2.09 (6.76 μM)	3.50 (15.08 μM)	3.24 (13.96 μM)
* half-life plasma (h)	4.4	7.1	4.2	5.3
* half-life tumour (h)	4.5	5.0	3.6	4.9

Legend: AUC, area under the curve; C_max_, maximal concentration. * Approximately estimated due to limited time points available.

## Data Availability

The data presented in this study are available on request from the corresponding author. The data are not publicly available due to privacy.

## Supplementary Materials

The following supporting information can be downloaded at: https://www.mdpi.com/article/10.3390/molecules28248056/s1, Figure S1: Chemical structures of ZST316 and L-257 (panel A) and proposed mechanism of conversion of ZST316 to L-257 by amidases (panel B); Table S1: Limit of detection (LOD) for the assay of ZST316 and L-257 in plasma; Table S2: Plasma concentrations of ZST316 after acute (single dose) treatment (i.p. 30 mg/Kg); Table S3: Plasma concentrations of L-257 after acute (single dose) treatment with ZST316 (i.p. 30 mg/Kg); Table S4: Tumour concentrations of ZST316 after acute (single dose) treatment (i.p. 30 mg/Kg); Table S5: Tumour concentrations of L-257 after acute (single dose) treatment with ZST316 (i.p. 30 mg/Kg); Table S6: Plasma concentrations of ZST316 after chronic (five-day) treatment (i.p. 30 mg/Kg daily); Table S7: Plasma concentrations of L-257 after chronic (five-day) treatment with ZST316 (i.p. 30 mg/Kg daily); Table S8: Tumour concentrations of ZST316 after chronic (five-day) treatment (i.p. 30 mg/Kg daily); Table S9: Tumour concentrations of L-257 after chronic (five-day) treatment with ZST316 (i.p. 30 mg/Kg daily); Table S10: Characteristics of the old [31] and new method.
