# Supplementary material for: Development of a HPLC-MS/MS Method to Assess the Pharmacokinetics and Tumour Distribution of the Dimethylarginine Dimethylaminohydrolase 1 Inhibitors ZST316 and L-257 in a Xenograft Model of Triple-Negative Breast Cancer in Mice
Source: Molecules. 2023 Dec 13;28(24):8056. doi: 10.3390/molecules28248056 (PMC10746103; doi:10.3390/molecules28248056)

Supplementary Table 1. Limit of detection (LOD) for the assay of ZST316 and L-257 in plasma.

**ZST316**			**L-257**		
Concentration	Peak Area	Signal to Noise	Concentration	Peak Area	Signal to Noise
2ng/mL	1360	3.7	2ng/mL	1720	3.4
	1260	2.8		1750	4.2
	1070	3.3		1710	3.3
Mean	1230	3.3	Mean	1727	3.6
SD	147	0.5	SD	21	0.5
CV	12.0%	13.8%	CV	1.2%	13.6%

Supplementary Table 2. Plasma concentrations of ZST316 after acute (single dose) treatment (i.p. 30 mg/Kg).

**# mice**	**time (h)**	**ZST316 (ng/mL)**	**Mean (ng/mL)**	**SD**	**CV%**	**ZST316 (μM)**	**Mean (μM)**
401	1	3000	2787	561.3	20.1%	9.70	9.01
409	1	2150				6.95	
419	1	3210				10.38	
423	8	119	143	21.8	15.3%	0.38	0.46
405	8	162				0.52	
408	8	147				0.48	
417	24	64.2	48	20.3	42.6%	0.21	0.15
430	24	53.7				0.17	
428	24	25				0.08	

Legend: SD, standard deviation; CV, coefficient of variation.

Supplementary Table 3. Plasma concentrations of L-257 after acute (single dose) treatment with ZST316 (i.p. 30 mg/Kg).

# mice	time (h)	L-257 (ng/mL)	Mean (ng/mL)	SD	CV%	L-257 (μM)	Mean (μM)
401	1	2610	2023	509.0	25.2%	11.24	8.72
409	1	1700				7.32	
419	1	1760				7.58	
423	8	502	385	115.0	29.9%	2.16	1.66
405	8	272				1.17	
408	8	382				1.65	
417	24	38	39	7.8	19.7%	0.16	0.17
430	24	32.5				0.14	
428	24	47.8				0.21	

Legend: SD, standard deviation; CV, coefficient of variation.

Supplementary Table 4. Tumour concentrations of ZST316 after acute (single dose) treatment (i.p. 30 mg/Kg).

# mice	time (h)	ZST316 (ng/g)	Mean (ng/g)	SD	CV%	ZST316 (µM)	Mean (µM)
401	1	2470	2000	688.3	34.4	7.99	6.47
409	1	1210				3.91	
419	1	2320				7.50	
423	8	149	144	32.3	22.5	0.48	0.46
405	8	109				0.35	
408	8	173				0.56	
417	24	37.3	39.5	8.6	21.8	0.12	0.13
430	24	32.2				0.10	
428	24	49.0				0.16	

Legend: SD, standard deviation; CV, coefficient of variation.

Supplementary Table 5. Tumour concentrations of L-257 after acute (single dose) treatment with ZST316 (i.p. 30 mg/Kg).

# mice	time (h)	L-257 (ng/g)	Mean (ng/g)	SD	CV%	L-257 (µM)	Mean (µM)
401	1	3590	3497	645.1	18.4	15.5	15.1
409	1	2810				12.1	
419	1	4090				17.6	
423	8	651	645	417.5	64.7	2.8	2.78
405	8	225				0.97	
408	8	1060				4.57	
417	24	30.7	40	14.0	35.1	0.13	0.17
430	24	33.1				0.14	
428	24	56.1				0.24	

Legend: SD, standard deviation; CV, coefficient of variation.

Supplementary Table 6. Plasma concentrations of ZST316 after chronic (five-day) treatment (i.p. 30 mg/Kg daily).

**# mice**	**time (h)**	**ZST316 (ng/mL)**	**Mean (ng/mL)**	**SD**	**CV%**	**ZST316 (µM)**	**Mean (µM)**
411	1	2440	2223	206.0	9.3%	7.89	7.19
407	1	2030				6.57	
404	1	2200				7.12	
426	8	81.7	59.4	20.4	34.3%	0.26	0.19
427	8	54.7				0.18	
425	8	41.8				0.14	
435	24	101	120.1	46.4	38.6%	0.33	0.39
410	24	173				0.56	
421	24	86.3				0.28	

Legend: SD, standard deviation; CV, coefficient of variation.

Supplementary Table 7. Plasma concentrations of L-257 after chronic (five-day) treatment with ZST316 (i.p. 30 mg/Kg daily).

# mice	time (h)	L-257 (ng/mL)	Mean (ng/mL)	SD	CV%	L-257 (µM)	Mean (µM)
411	1	1600				6.89	
407	1	1500	1603	105.0	6.6%	6.46	6.91
404	1	1710				7.37	
426	8	209				0.90	
427	8	249	198	57.3	28.9%	1.07	0.85
425	8	136				0.59	
435	24	58.8				0.25	
410	24	95.2	61	33.1	54.2%	0.41	0.26
421	24	29.1				0.13	

Legend: SD, standard deviation; CV, coefficient of variation.

Supplementary Table 8. Tumour concentrations of ZST316 after chronic (five-day) treatment (i.p. 30 mg/Kg daily).

# mice	time (h)	ZST316 (ng/g)	Mean (ng/g)	SD	CV%	ZST316 (µM)	Mean (µM)
411	1	2490				8.05	
407	1	1750	2087	374.5	17.9	5.66	6.75
404	1	2020				6.53	
426	8	120				0.39	
427	8	110	125	18.6	14.8	0.36	0.41
425	8	146				0.47	
435	24	37.8				0.12	
410	24	108	57.0	44.6	78.3	0.35	0.18
421	24	25.2				0.08	

Legend: SD, standard deviation; CV, coefficient of variation.

Supplementary Table 9. Tumour concentrations of L-257 after chronic (five-day) treatment with ZST316 (i.p. 30 mg/Kg daily).

# mice	time (h)	L-257 (ng/g)	Mean (ng/g)	SD	CV%	L-257 (µM)	Mean (µM)
411	1	3550				15.29	
407	1	2810	3237	382.8	11.8	12.10	13.94
404	1	3350				14.43	
426	8	413				1.78	
427	8	403	381	47.6	12.5	1.74	1.64
425	8	326				1.40	
435	24	35.5				0.15	
410	24	159	97	87.3	89.8	0.68	0.30
421	24	<LOQ				0.05	

Legend: SD, standard deviation; CV, coefficient of variation; LOQ, limit of quantification.

Supplementary Table 10. Characteristics of the old [31] and new method.

<table>
<tr><th></th><th>Matrices analyzed</th><th>Compounds analyzed</th><th>Similarities</th><th>Differences</th><th>Improvements with the new method</th></tr>
<tr><td>Old method [31]</td><td>Plasma</td><td>ZST316
ZST152 (IS)</td><td rowspan="2">Chromatographic characteristics (MP, column, gradient features, and duration)

Extractive solvent and handling procedures

Sensitivity in plasma (LOQ = 5ng/mL)</td><td>MS/MS ionization mode: negative

Calibration plasma range: 5-500 ng/mL

Target ions m/z 208.0 for ZST316 and m/z 170.9 for IS</td><td rowspan="2">Wider range of linearity reduces the need for dilutions.

The simultaneous measurement of the two molecules allows a more comprehensive pharmacokinetic analysis and limits the amount of matrix needed.</td></tr>
<tr><td>New method</td><td>Plasma and tumour</td><td>ZST316
L-257
ZST152 (IS)</td><td>MS/MS ionization mode: negative

Calibration plasma range: 5-1000 ng/mL

Target ions m/z 115.3 for ZST316 and m/z 256.2 for IS</td></tr>
</table>

Legend: IS, internal standard; LOQ, limit of quantification; MP, mobile phase; MS/MS, tandem mass spectrometry; m/z, mass-to-charge ratio.

Supplementary Figure 1

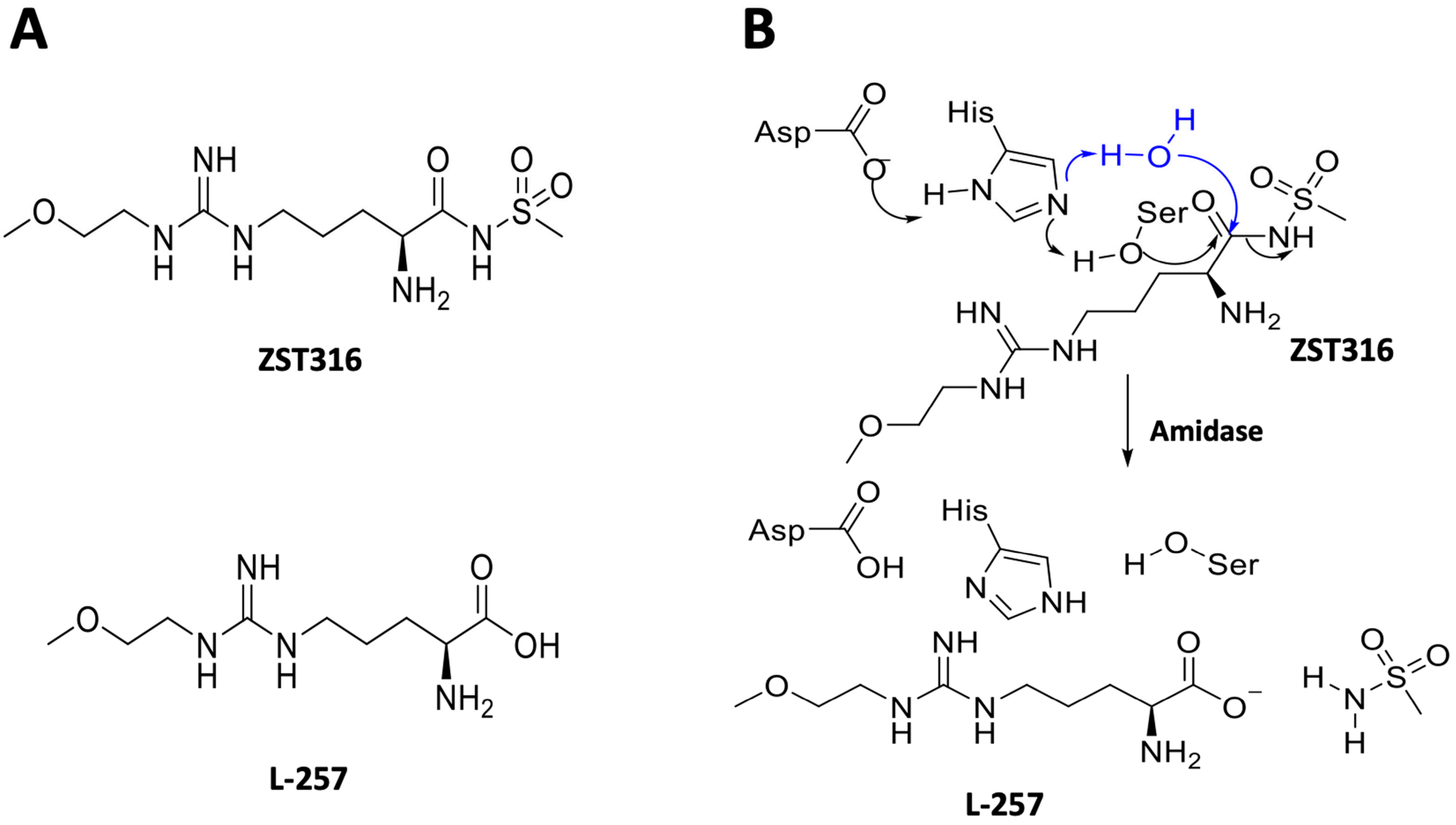

Supplement: Supplementary file 1 [file molecules-28-08056-s001.zip › molecules-2682231-supplementary.pdf]
